# Household Clusters of Severe Acute Respiratory Syndrome Coronavirus 2 (SARS-CoV-2) Infection in Suzhou, China

**DOI:** 10.1155/2021/5565549

**Published:** 2021-10-16

**Authors:** Lin Yao, Peijun Tang, Hui Jiang, Binbin Gu, Ping Xu, Xiafang Wang, Xin Yu, Jianping Zhang, Yu Pang, Meiying Wu

**Affiliations:** ^1^Department of Pulmonary, The Fifth People's Hospital of Suzhou, The Affiliated Infectious Diseases Hospital of Soochow University, Suzhou 215000, China; ^2^Department of Bacteriology and Immunology, Beijing Key Laboratory on Drug-Resistant Tuberculosis Research, Beijing Chest Hospital, Capital Medical University/Beijing Tuberculosis & Thoracic Tumor Research Institute, Beijing 101149, China; ^3^Intensive Care Unit, The Fifth People's Hospital of Suzhou, The Affiliated Infectious Diseases Hospital of Soochow University, Suzhou 215000, China; ^4^Department of Clinical Laboratory, The Fifth People's Hospital of Suzhou, The Affiliated Infectious Diseases Hospital of Soochow University, Suzhou 215000, China

## Abstract

**Objectives:**

The severe acute respiratory syndrome coronavirus 2 (SARS-CoV-2) is an emerging virus causing substantial morbidity and mortality worldwide. We performed a cross-sectional investigation of SARS-CoV-2 clusters in Suzhou to determine the transmissibility of the virus among close contacts and to assess the demographic and clinical characteristics between index and secondary cases.

**Methods:**

We review the clustered patients with SARS-CoV-2 infections in Suzhou between 22 January and 29 February 2020. The demographic and clinical characteristics were compared between index and secondary cases. We calculated the basic reproduction number (*R*_0_) among close contacts with SLI model.

**Results:**

By 22 February, 87 patients with SARS-CoV-2 infection were reported, including 50 sporadic and 37 clustered cases, who were generated from 13 clusters. On admission, 5 (20.8%) out of 24 secondary cases were asymptomatic. The male ratio of index cases was significantly higher than that of secondary cases. Additionally, the index cases were more likely to have fever and increased CRP levels than the secondary cases. The *R*_0_ values of clusters displayed a significantly declining trend over time for all clusters. The relative risk of infection in blood-related contacts of cases versus unrelated contacts was 1.60 for SARS-CoV-2 (95% CI: 0.42-2.95).

**Conclusions:**

In conclusion, SARS-CoV-2 has great person-to-person transmission capability among close contacts. The secondary cases are more prone to have mild symptoms than index cases. There is no increased RR of secondary infection in blood relatives versus unrelated contacts. The high rate of asymptomatic SARS-CoV-2 infections highlights the urgent need to enhance active case finding strategy for early detection of infectious patients.

## 1. Introduction

Since December 2019, China has been experiencing the emergence of a novel coronavirus, named as SARS-CoV-2, which can cause human coronavirus disease-19 (COVID-19), mainly presenting with respiratory disease, severe pneumonia, and multiple organ damage [1]. Although the origin of the infections has yet to be identified to date, the SARS-CoV-2 is capable of human-to-human transmission [2]. Although there is occasional amplification in the healthcare settings, the predominant transmission potential of SARS-CoV-2 occurs in the persons who have unprotected exposure to confirmed or subclinical cases [3]. Therefore, epidemiological analyses of detailed line lists of patients are of great importance to identify key parameters for understanding its infectivity, which is essential to formulate effective measures to protect close contacts against secondary infection.

The transmissibility of SARS-CoV-2 is the most important determinant of pandemic crisis and has become worrisome given the community outbreaks consisting of clusters with 2 or more epidemiologically linked cases [4]. Previous studies that have modeled the reproductive rate of SARS-CoV-2 in human have been based on the notified and clinically apparent cases from epidemiological investigations or official websites on COVID-19 [1, 5, 6]. However, the exclusion of individuals with no or only mild respiratory symptoms becomes the barrier to conduct in-depth investigation of rate of secondary transmission in clusters. In addition, the meticulous analysis of clusters provides insights into not only the transmissibility of SARS-CoV-2 but also the familial susceptibility [4, 7]. To address this concern, we performed a cross-sectional investigation of 13 SARS-CoV-2 clusters in Suzhou to determine the transmissibility of the virus among close contacts. The second objective of this study was to assess the demographic and clinical characteristics between index and secondary cases.

## 2. Methods

### 2.1. Patients

This study was conducted in the Fifth People's Hospital of Suzhou, which is a government-designated hospital for emerging infectious diseases. There have been 87 cases with laboratory-confirmed SARS-CoV-2 infections between 22 January and 29 February 2020, including 50 sporadic and 37 clustered cases. Laboratory-confirmed testing for SARS-CoV-2 infection was conducted using quantitative RT-PCR (qRT-PCR) by detection of SARS-CoV-2-specific fragment. The 37 patients associated with the 13 index patients were further included for epidemiological analysis. The routine blood counts and biochemical tests were assessed at the same day postsymptom onset by the Clinical Laboratory of the hospital. Demographic, clinical, and laboratory characteristics were obtained with data collection forms from electronic medical records. This study was approved by the Ethics Committee of the Fifth People's Hospital of Suzhou (No. 2020SZWY005). Written consent was obtained from each participant.

### 2.2. Definitions

All cases were diagnosed according to the detection SARS-CoV-2 in throat, nasopharyngeal, or cloacal swabs regardless of disease severity. The contacts were defined as individuals living in the same house or working in the same office. The blood-relative relationship was defined as parent-offspring, siblings, grandparent-grandchild, and uncle/aunt-niece/nephew, whereas the unrelated contact was defined as spouse, son/daughter-in-law, parent-in-law, workmate, and the other unrelated member [4]. We defined the index patient as the person with the earliest symptom onset date or laboratory-confirmed COVID-19 plus exposure in Wuhan.

### 2.3. Statistical Analysis

Chi-square or Fisher exact test was used, as appropriate, to compare continuous variables, which were presented with frequency (percentage). Continuous variables were summarized as means and standard deviations (SD), and the student *t*-test was used for comparison. *P* values of < 0.05 were considered statistically significantly. All calculations were carried out using SPSS 20.0 (SPSS Inc., Chicago, IL, USA).

In order to investigate whether there is genetic susceptibility to COVID-19, the data from the clustered cases were used to calculate the relative risk (RR) of infection of blood relatives versus unrelated contacts of the index case based on the assumption that the blood relatives had the same probability of detection infection as unrelated contacts. We followed the method used by Lemaitre and colleague [4]. In order to calculate the basic reproduction number (*R*_0_) among close contacts, we proposed the following SLI model [8]. The secondary attack rate was calculated as the number of secondary cases divided by the total number of household contacts. All analyses were conducted in the R software environment for statistical computing.

## 3. Results

### 3.1. Study Population

**Figure 1:**
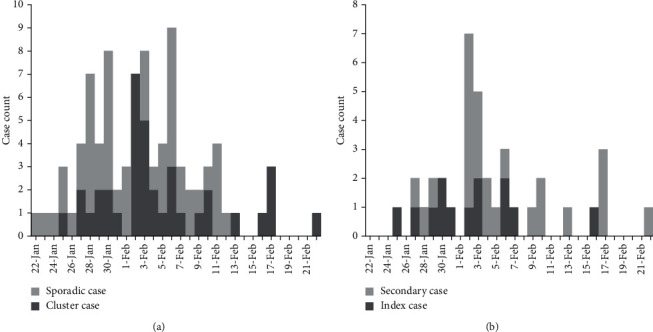
Cases of laboratory-confirmed SARS-CoV-2 infection reported between 22 January and 29 February 2020.

On 22 January, the first laboratory-confirmed SARS-CoV-2 case in Suzhou was reported in a 37-year-old male who returned from Wuhan. By 22 February, 87 patients with SARS-CoV-2 infection were reported, including 50 (57.5%) sporadic and 37 (42.5%) clustered cases (Figure 1). The 37 clustered cases were generated from 13 clusters. The clusters included a mean of 4.8 contacts (range, 2 to 12). Of 13 clusters, 12 were household contacts, and the remaining one cluster was workmate contact. The median age of the 13 index patients was 49 years (range, 30 to 64 years). Of 37 patients, 20 (54.0%) were male. Five (13.5%) of the patients had comorbidities (Table S1).

### 3.2. Clinical Characteristics

On admission, 32 patients had clinical symptoms or signs, and the other 5 patients were asymptomatic, who had positive qRT-PCR results by testing all close contacts of confirmed cases. Of 32 patients with clinical symptoms, 23 (71.9%) had fever or cough, and gastrointestinal symptoms (vomiting, nausea, and diarrhea) were prominent in five (15.6%) patients. For blood counts, 36 (97.3%) patients had normal or lower than average total white blood cell counts, and 24 (64.9%) had normal or lower than average lymphocyte cell counts. By contrast, 16 (43.2%) patients had substantially increased C-reactive protein levels. In addition, the increased procalcitonin levels were observed in 7 (18.9%) patients. All patients were treated in isolation. The durations for antiviral treatment and antibiotics treatment were 2-28 days (median 5.5 days) and 3-21 days (median 12.5 days), respectively.

### 3.3. Index and Secondary Cases

The demographic and clinical characteristics of patients are indicators for viral virulence. In order to examine whether the virulence of SARS-CoV-2 varied during human-to-human transmission, we compared the individual characteristics between index and secondary clustered cases. As shown in Table 1, the average age of index cases was higher than secondary cases (median age 49 years vs. 36; *P* = 0.017). The male ratio of index cases was significantly higher than that of secondary cases (*P* = 0.040). Additionally, the index cases were more likely to have fever than the secondary cases (92.3% vs. 62.3%; *P* = 0.015). The interval between the onset of symptoms and final diagnosis for index cases (median 6 days) was longer than that for secondary cases (median 4 days), but the difference was not statistically significant (*P* = 0.098).

We further assessed the difference in blood routine and biochemical tests between index and secondary cases. As summarized in Table 2, the index cases were more likely to have increased CRP levels than secondary cases (23.48 mg/L vs. 7.91; *P* = 0.003). For biochemical test, the average creatinine level of index cases (68.20 *μ*mol/L) was significantly higher than that of secondary cases (52.48 *μ*mol/L), whereas the index cases (35.85 g/L) had lower level of albumin compared with the secondary cases (38.98 g/L; *P* = 0.002).

### 3.4. Epidemiological Characteristics of SARS-CoV-2 in Clusters

**Figure 2:**
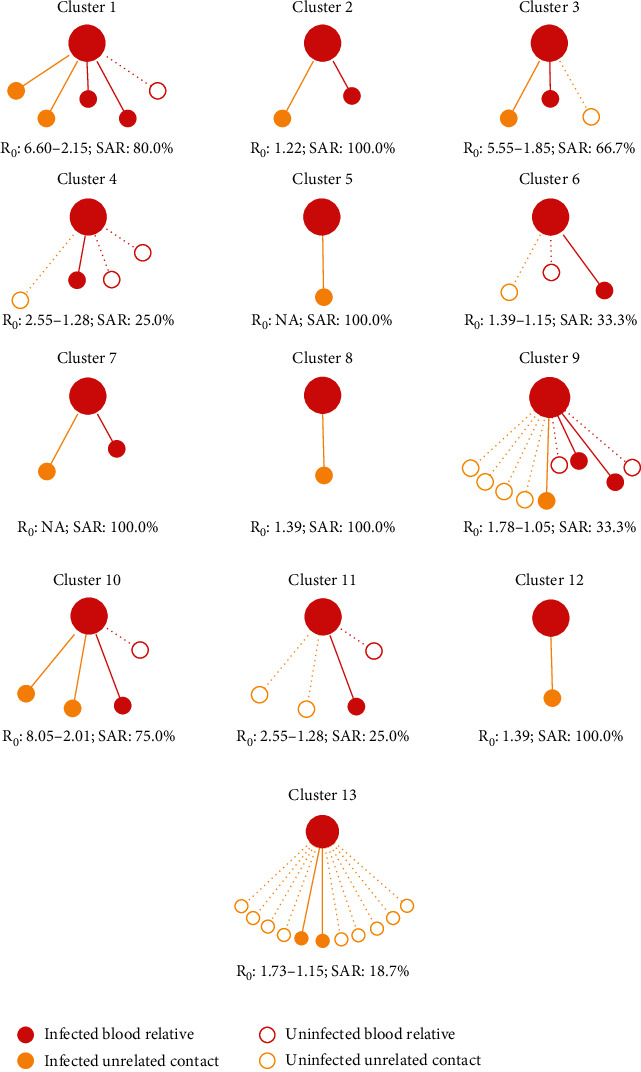
Transmission of reported clusters of SARS-CoV-2 infections as differentiated by blood relatives and unrelated contacts. SAR: secondary attract rate. The red solid circle represents infected blood relatives. The red hollow circle represents uninfected blood relatives. The yellow solid circle represents infected unrelated contacts. The yellow hollow circle represents uninfected unrelated contacts. The distance between index and secondary case represents kin relationship.

Figure 2 summarizes the epidemiological characteristics of SARS-CoV-2 clusters in our cohort. Overall, the *R*_0_ values of clusters displayed a significantly declining trend over time for all clusters. The highest *R*_0_ was observed in early stage of transmission in Cluster 10, with a *R*_0_ value of 8.05, followed by 6.60 in Cluster 1 and 5.55 in Cluster 3. At the end stage of transmission, each *R*_0_ value ranged from 1.05 to 2.15 across clusters. Using full data on close contacts, the RR of infection in blood-related contacts of cases versus unrelated contacts was 1.60 for SARS-CoV-2 (95% CI: 0.42-2.95).

## 4. Discussion

The outbreak of SARS-CoV-2 has triggered a rising global health emergency [9, 10]. To our knowledge, this is the first report on its epidemiological characteristics among close contacts. Our results demonstrate that SARS-CoV-2 has great person-to-person transmission capability; however, the high *R*_0_ values are only observed in early stage rather than the later stage of transmission. The substantial decline of *R*_0_ values majorly attributes to limited contact of index and secondary cases in the community. Notably, the most important risk factor for acquiring SARS-CoV-2 infection in our clusters is close household contacts because of their prolonged exposure to the index cases in their house, which are relatively confined and poorly ventilated airspaces [11]. In addition, the adoption of strict precautions, such as closed community and delayed resumption of work [12], is essential to prevent its transmission in the workplace. Despite limited spread in household contacts, it would lead to exponential increase in burden of COVID-19. Therefore, we speculate that the isolation of the COVID-19 cases in so-called shelter or “Fang Cang” hospitals rather than the recommendation of cases with mild symptoms to be cared for at home by family members in Wuhan since early February has played an important role in the decline in new infections among household contacts, thereby accelerate control of SARS-CoV-2 epidemic.

Another interesting finding of this study is that the secondary COVID-19 cases are more prone to have mild symptoms than index cases. Symptomatic index cases are more likely to seek medical health care, thereby triggering active identification of secondary cases with mild symptoms. This phenomenon may be majorly associated with a higher likelihood of detecting asymptomatic secondary cases when actively contact tracing. In addition, previous studies have demonstrated that the loss in virulence that occurs following transmission may have implications for the capability of viruses to transmit disease [13, 14]. Of note, as a new coronavirus of probable bat origin [15], SARS-CoV-2 crosses into new host population, and the continual introduction of new selective pressures would accumulate the accumulation of deleterious mutation conferring reduced virulence [16, 17]. Further comparative genomic analysis of viruses isolated from index and secondary cases will bring new insights into molecular mechanism involving the loss of virulence during transmission.

We have shown that the ratio of male to female in secondary cases is markedly lower than that in index cases. Although this suggests a potential increased susceptibility to SARS-CoV-2 infections for female in clusters, the expression level of ACE2, the cell entry receptor of this novel coronavirus, shows no significant difference between gender groups [18, 19], which is opposed to our finding. After a careful analysis of the cases, a plausible explanation is that the high rate of male index case is associated with the gender-related change in secondary cases considering that the household contacts at the highest risk of subsequent infections are spouses due to their prolonged intimate contact. This hypothesis is supported by our finding that no increased RR of secondary infection in blood relatives is found compared with unrelated contacts. This epidemiological parameter is consistent with that of H7N9 but different from that of H5N1 [4]. More evidence is urgently needed to elucidate the varying host susceptibility across viruses.

Notably, approximate one-fifth of secondary cases in clusters are asymptomatic infections, which is higher than the proportion in SARS-CoV (13%) [20]. The substantially high proportion of asymptomatic SARS-CoV-2 infections may be an indicator for reduced virulence than SARS-CoV, which corroborates previous findings that the mortality rate in patients with SARS-CoV-2 infection is lower than that previously seen in SARS patients [21, 22]. In a recent case series, asymptomatic persons are potential sources of SARS-CoV-2 infections [23]. In this context, despite the lack of knowledge on the contribution of asymptomatic persons to transmission, the control measures will be hampered since they depend on the screening strategy of patients with clinical symptoms.

This study has several obvious limitations. First, despite enrolment of all clusters in Suzhou, the small sample size of our study necessitates cautious interpretation of our findings. Second, molecular assays currently available have low sensitivity during the initial stage of infection [24]. In view of the laboratory-confirmed cases included in this study, the exclusion of false-negative cases may lead to the underestimation of basic reproductive number. Finally, the genome sequence of each virus was not analyzed due to the biosafety risk concern. Hence, we could not identify nucleotide variations during transmission. Nevertheless, this first report on epidemiological characteristic of SARS-CoV-2 infections in clusters will help us to take effective actions to curb its transmission in close contacts.

In conclusion, our results demonstrate that SARS-CoV-2 has great person-to-person transmission capability; however, the high *R*_0_ values are only observed in early stage rather than later stage of transmission. The secondary COVID-19 cases are more prone to have mild symptoms than index cases, which may be majorly associated with a higher likelihood of detecting asymptomatic secondary cases when actively contact tracing. There is no increased RR of secondary infection in blood relatives versus unrelated contacts. The high rate of asymptomatic SARS-CoV-2 infections highlights that the control measures will be hampered since they depend on the screening strategy of patients with clinical symptoms.

## Figures and Tables

**Table 1:** Demographic and clinical characteristics of index and secondary cases in clusters.

Characteristic	Total (*N* = 37, %)	Cluster index cases (*n* = 13, %)	Cluster secondary cases (*n* = 24, %)	*P* value
Age (median, IQR)		49 (39.5-61.5)	36 (22.5-46.8)	0.017
Age group				0.054
0-14	5 (13.5)	0 (0.0)	5 (20.8)	
15-24	2 (5.4)	0 (0.0)	2 (8.3)	
25-59	23 (62.2)	9 (69.2)	14 (58.3)	
≥60	7 (18.9)	4 (30.8)	3 (12.5)	
Gender				0.040
Female	17 (45.9)	3 (23.1)	14 (58.3)	
Male	20 (54.1)	10 (76.9)	10 (41.7)	
Outcome				0.260
Survive	21 (56.8)	9 (69.2)	12 (50.0)	
In treatment	16 (43.2)	4 (30.8)	12 (50.0)	
Underlying chronic medical conditions				
No	32 (86.5)	11 (84.6)	21 (87.5)	0.808
Cardiovascular disease	1 (2.7)	0 (0.0)	1 (4.2)	1.000
Diabetes mellitus	1 (2.7)	1 (7.7)	0 (0.0)	0.351
Hypertension	1 (2.7)	1 (7.7)	0 (0.0)	0.351
Others	2 (5.4)	0 (0.0)	2 (8.3)	0.532
Onset symptom				
Fever	23 (62.2)	12 (92.3)	11 (45.8)	0.015
Cough	23 (62.2)	11 (84.6)	12 (50.0)	0.086
Sore throat	3 (8.1)	1 (7.7)	2 (8.3)	1.000
Runny nose	3 (8.1)	0 (0.0)	3 (12.5)	0.485
Sputum production	17 (45.9)	8 (61.5)	9 (37.5)	0.161
Vomiting	1 (2.7)	1 (7.7)	0 (0.0)	0.351
Nausea	1 (2.7)	1 (7.7)	0 (0.0)	0.351
Fatigue	6 (16.2)	2 (15.4)	4 (16.7)	1.000
Sore muscles	1 (2.7)	0 (0.0)	1 (4.2)	1.000
Diarrhea	3 (8.1)	1 (7.7)	2 (8.3)	1.000
Clinical course, median (IQR), days				
From illness onset to diagnosis	4.5 (2.3-8.8)	6 (4-13)	4 (4-7)	0.098
From diagnosis to cured	16 (12-19)	20 (11.5-25.5)	15 (12-19)	0.511
Duration of antiviral treatment				
Median days (IQR)	11 (8-15)	12 (8-16)	11 (8-14)	0.122
Duration of antibiotics treatment				
Median days (IQR)	13 (10-17)	15 (12-20)	10 (3-14)	0.001
Duration of hormone treatment				
Median days (IQR)	0 (0-3)	0 (0-5)	0 (0-0)	—
Complication				
No	32 (86.5)	11 (84.6)	21 (87.5)	
Respiratory failure	4 (10.8)	2 (15.4)	2 (8.3)	0.742
Other type of pneumonia	1 (2.7)	0 (0.0)	1 (4.2)	0.838

**Table 2:** Results of laboratory tests of index and secondary cases in clusters.

Characteristic	Total (*N* = 37, *x* ± *s*)	Cluster index cases (*n* = 13, *x* ± *s*)	Cluster secondary cases (*n* = 24, *x* ± *s*)	*P* value
Infection-related biomarker				
C-reaction protein (mg/L)	13.37 ± 16.04	23.48 ± 18.89	7.91 ± 11.28	0.003
Blood biochemical test				
Albumin (g/L)	37.88 ± 3.11	35.85 ± 2.81	38.98 ± 2.72	0.002
Creatinine (*μ*mol/L)	58.00 ± 21.26	68.20 ± 19.02	52.48 ± 20.69	0.030

## Data Availability

The datasets generated and analyzed from the current study are not publicly available at this time as further analyses are ongoing but are available from the corresponding author on reasonable request.

## Abbreviations

SARS-CoV-2: Severe acute respiratory syndrome coronavirus 2
COVID-19: Coronavirus disease-19
R0: Basic reproduction number
SD: Standard deviations
RR: Relative risk.

## Supplementary Materials

Supplementary Materials Supplemental Table 1: demographic and clinical characteristics of laboratory-confirmed cases with SARS-CoV-2 infection in clusters.
