# Supplementary material for: Household Clusters of Severe Acute Respiratory Syndrome Coronavirus 2 (SARS-CoV-2) Infection in Suzhou, China
Source: Biomed Res Int. 2021 Oct 16;2021:5565549. doi: 10.1155/2021/5565549 (PMC8520496; doi:10.1155/2021/5565549)
Supplement: Supplementary Materials — Supplemental Table 1: demographic and clinical characteristics of laboratory-confirmed cases with SARS-CoV-2 infection in clusters. [file 5565549.f1.pdf]

**Cluster ID**	**Province**	**Patient ID in cluster**	**Age**	**Sex**	**Urban/Rural**	**Relationship to index case**	**Time of onset of illness**
1	Hubei	1	63	Male	Urban	Index case	2020-01-25
1	Jiangsu	2	64	Female	Urban	Spouse	2020-01-26
1	Hubei	3	38	Male	Urban	Son	2020-01-31
1	Shanxi	4	37	Female	Urban	Daughter-in-law	2020-02-01
1	Hubei	5	9	Female	Urban	Granddaughter	2020-01-27
2	Hubei	1	60	Male	Urban	Index case	2020-01-17
2	Jiangsu	2	49	Female	Urban	Spouse	2020-01-27
2	Jiangsu	3	24	Male	Urban	Son	2020-01-24
3	Hubei	1	55	Male	Urban	Index case	2020-01-28
3	Hubei	2	35	Male	Urban	Son	2020-01-31
3	Hubei	2	33	Female	Urban	Daughter-in-law	2020-02-01
**4**	Jiangsu	1	36	Male	Urban	Index case	2020-01-24
**4**	Jiangsu	2	77	Male	Urban	Father	Asystomatic
**5**	Hubei	1	43	Male	Urban	Index case	2020-01-26
**5**	Hubei	2	43	Female	Urban	Spouse	2020-01-29
**6**	Liaoning	1	63	Female	Urban	Index case	2020-01-20
**6**	Jiangsu	2	11	Female	Urban	Grandson	2020-01-29
**7**	Heilongjiang	1	47	Male	Urban	Index case	2020-01-27
**7**	Heilongjiang	2	46	Female	Urban	Spouse	2020-02-01
**7**	Heilongjiang	3	22	Female	Urban	Daughter	2020-01-28
**8**	Hubei	2	64	Male	Urban	Spouse	2020-01-28
**8**	Hubei	1	64	Female	Urban	Index case	2020-01-22
**9**	Henan	1	30	Male	Urban	Index case	2020-01-28
**9**	Henan	2	54	Male	Urban	Father	2020-02-05
**9**	Henan	3	29	Female	Urban	Spouse	2020-02-06
**9**	Henan	4	4	Male	Urban	Grandson	Asystomatic
**10**	Hubei	1	49	Male	Urban	Index case	2020-02-14
**10**	Hubei	2	47	Female	Urban	Spouse	Asystomatic
**10**	Hubei	3	27	Female	Urban	Daughter-in-law	Asystomatic
**10**	Hubei	4	1	Female	Urban	Granddaughter	Asystomatic
**11**	Hubei	1	57	Female	Urban	Index case	2020-01-26
**11**	Jiangsu	2	1	Male	Urban	Grandson	2020-01-27
**12**	Jiangsu	1	47	Male	Urban	Index case	2020-01-26
**12**	Hubei	2	44	Female	Urban	Spouse	2020-02-01
**13**	Jiangsu	1	45	Male	Urban	Index case	2020-01-29
**13**	Jiangsu	2	25	Male	Rural	Workmate	2020-01-30
**13**	Jiangsu	3	34	Male	Urban	Workmate	2020-01-29

Confirmed time	WBC	HGB	PLT	Neutrophils	Leucocytes	CRP	PCT	TBil
2020-02-03	3.1	149	111	1.45	1.12	41.8	0.034	15.1
2020-02-03	6.74	126	156	5.26	1.03	34.4	0.043	11
2020-02-04	3.39	130	169	1.81	0.89	1.2	0.029	7.1
2020-02-04	3.36	138	212	1.65	1.32	18	0.02	7
2020-02-03	8.04	132	318	5.12	2.18	0.5	0.028	4.5
2020-01-25	6.06	148	124	3.69	1.63	23.6	0.044	9.1
2020-01-27	4.17	156	227	2.76	1.05	1.5	<0.020	7.7
2020-01-29	8.24	170	262	6.06	1.45	0.5	0.044	11.2
2020-02-06	6.13	140	259	3.3	2.07	0.5	0.02	6
2020-02-02	1.68	140	92	0.63	0.81	17.6	0.071	6
2020-02-02	3.23	123	82	2.4	0.58	20.4	0.028	3.5
2020-01-30	6.99	151	370	3.53	2.44	1	0.021	16.2
2020-02-10	4.72	134	272	2.57	1.34	0.5	0.076	10.1
2020-01-30	5.19	135	119	2.62	1.66	2.8	0.037	9.7
2020-02-02	5.57	123	123	3.82	1.2	31	0.022	15.1
2020-01-31	6.11	135	195	4.42	1.16	37.9	1.1	8.6
2020-02-02	9.1	143	261	5.3	3.22	0.5	0.026	7.1
2020-02-02	3.72	148	115	2.24	1.09	23.8	0.043	11.1
2020-02-02	3	137	166	1.69	1.08	22.6	0.02	8.9
2020-02-02	4.48	155	188	2.36	1.59	2.7	0.02	9
2020-02-03	5.9	139	190	4.35	0.99	5.9	0.026	17.7
2020-01-29	5.12	148	152	3.17	1.36	16.2	0.068	10
2020-02-03	5.12	160	115	3.62	0.97	24.6	0.032	5.3
2020-02-05	7.16	147	207	3.54	2.77	0.5	0.029	4.6
2020-02-06	6.41	126	210	3.77	2.26	0.5	0.02	19.3
2020-02-22	8.14	127	249	3.56	3.68	0.5	0.099	6.2
2020-02-16	3.22	132	167	2.09	0.85	16.3	0.036	18.1
2020-02-17	6.16	96	263	4.7	0.81	1.9	0.022	6.5
2020-02-17	4.27	131	123	1.92	1.92	<0.5	<0.020	8.5
2020-02-17	6.22	128	287	0.76	4.92	<0.5	0.025	4.8
2020-01-27	6.92	121	179	4.65	1.75	49	0.021	10.4
2020-01-28	12.94	117	212	0.63	11.46	0.5	0.034	4.8
2020-02-07	4.9	143	142	3.82	0.68	59.4	0.108	10.8
2020-02-09	2.89	125	119	1.68	0.83	2.4	0.047	10.9
2020-02-06	9.06	155	307	6.31	1.9	8.3	<0.020	14.1
2020-02-10	7.39	162	208	5.46	1.44	0.5	0.02	14.3
2020-02-13	6.23	139	323	3.57	1.87	25.8	<0.020	16

**Albumin**	**AST**	**ALT**	**LDH**	**BUN**	**Cr**	**CK**	**N-proBNP**	**cTnT**
34.3	44	29	525	3.43	67.1	282	115	9
36	49	45	830	3.83	36.7	212	96	10
38.2	21	33	321	3.69	82.5	144	8	10
41.5	23	21	455	2.37	47.6	45	25	3
41.7	42	69	689	4.14	33.7	67	14	4
39.6	17	18	184	6.36	86.3	182	871	12
37	18	23	328	3.58	63.9	21	7	<3.00
38	15	36	356	5.39	103.7	45	42	4
38.3	20	46	362	3.86	38.9	44	62	3
36.7	82	89	990	5.16	67.9	609	5	4
38.8	27	23	435	2.93	58.3	41	90	3
35.8	33	38	505	4.56	92	49	35	5
38.9	28	48	342	4.72	49.6	37	18	7
37.8	32	35	343	5.12	95.4	38	<5.00	5
41.2	22	17	370	3.68	63.2	38	11	<3.00
33.4	30	35	592	3.94	33	23	58	3
44	24	41	423	5.32	43.4	29	5	3
32.8	32	29	804	6.09	88.9	128	16	6
40	22	21	460	3.02	46.3	45	41	3
38.5	21	22	381	3.09	59.2	32	21	3
32.8	26	26	229	2.66	53.8	57	27	9
34.5	43	49	540	3.68	66	56	5	4
31.6	28	33	423	3.91	57.9	20	11	<3.00
39.1	24	44	307	6.51	55.3	25	23	3
36.9	14	31	313	4.2	43.1	22	31	3
43	26	31	434	5.15	21.4	101	54	6
35	24	45	506	3	63.8	62	43	7
39.9	15	25	349	3.44	44.9	24	59	<3.00
39.7	20	30	322	3.7	50.1	36	6	<3.00
38.5	55	53	637	3.78	16.9	84	169	6
41.3	38	34	626	4.93	61.4	70	43	7
39.2	49	24	558	4.63	14.2	82	319	5
34.6	32	29	360	4.1	66.3	137	78	13
38.3	21	19	254	2.66	52.8	74	23	5
37	86	271	732	8.37	69.6	25	17	<3.00
43.5	29	97	394	4.05	69	42	5	3
34.1	24	49	477	3.56	82	53	101	12

CK-MB	Myoglobin	Fever	Cough	Sputum production	Diarrhea	Vomiting	Nausea	Fatigue
3.04	46.77	1	1	1	0	1	1	0
3.32	59.01	0	0	0	1	0	0	1
0.94	36.03	1	1	0	0	0	0	0
1.02	21	0	1	1	0	0	0	0
1.68	21	0	1	0	0	0	0	0
1.73	46.39	1	1	0	0	0	0	0
<0.300	<21.00	1	1	1	0	0	0	0
0.41	29.53	1	1	1	0	0	0	0
0.49	21	0	1	0	0	0	0	0
0.38	101.3	1	1	1	0	0	0	0
0.3	21	1	0	0	0	0	0	0
0.61	21	1	1	1	0	0	0	0
0.96	21	0	0	0	0	0	0	0
0.34	<21.00	1	1	1	0	0	0	0
<0.300	24.54	1	0	0	0	0	0	0
0.42	21	1	1	0	0	0	0	0
0.32	21	0	1	1	0	0	0	0
0.51	47.16	1	1	1	0	0	0	0
0.46	21	0	1	1	0	0	0	0
0.3	21	0	1	1	0	0	0	0
1.19	25.73	1	1	0	0	0	0	1
0.6	23.59	1	1	1	0	0	0	0
<0.300	<21.00	1	0	0	1	0	0	1
0.56	21	1	0	0	0	0	0	0
0.56	21	0	0	0	0	0	0	1
5.18	<21	0	0	0	0	0	0	0
0.43	23.95	1	1	1	1	0	0	0
0.79	<21.00	0	0	0	0	0	0	0
0.6	<21.00	0	0	0	0	0	0	0
4.37	<21.00	0	0	0	0	0	0	0
<0.300	<21.00	1	0	0	0	0	0	0
2.58	21	1	0	0	0	0	0	0
0.98	44.81	1	1	1	0	0	0	1
<0.300	<21.00	1	0	0	0	0	0	0
0.45	26.8	1	1	1	0	0	0	1
0.41	21	1	1	1	0	0	0	0
0.42	28.81	0	1	1	0	0	0	0

**Runny nose**	**Sore muscles**	**Sore throat**
0	0	0
0	0	0
0	0	0
0	0	0
0	0	1
0	0	0
1	0	0
0	0	0
0	0	0
0	0	0
0	0	0
0	0	0
0	0	0
0	0	0
0	0	1
0	0	0
0	0	0
0	0	0
0	0	0
1	0	0
0	0	0
0	0	0
0	1	0
0	0	0
0	0	0
0	0	0
0	0	0
0	0	0
0	0	0
0	0	0
0	0	0
1	0	0
0	0	1
0	0	0
0	0	0
0	0	0
0	0	0
